# Synergy-based functional electrical stimulation and robotic-assisted for retraining reach-to-grasp in stroke: a study protocol for a randomized controlled trial

**DOI:** 10.1186/s12883-023-03369-2

**Published:** 2023-09-12

**Authors:** Huan-xia Zhou, Jun Hu, Rui-sheng Yun, Zhong-zhi Zhao, Ming-hui Lai, Li-hui-zi Sun, Kai-liang Luo

**Affiliations:** 1 Department of Rehabilitation Medical Center, Seventh People’s Hospital of Shanghai University of Traditional Chinese Medicine, Shanghai, China; 2 Department of Occupational Therapy, The Second Rehabilitation Hospital of Shanghai, No.25, Lane 860, Changjiang Road, Baoshan District, Shanghai, 200441 China; 3 Department of Mental Health Rehabilitation Center, Peking University Sixth Hospital, Beijing, China; 4 Department of Rehabilitation, The First Affiliated Hospital of Fujian Medical University, Fujian, China

**Keywords:** Stroke, Upper limb, Motor function, Synergy-based functional electrical stimulation, Robotic-assisted therapy

## Abstract

**Background:**

Stroke survivors have long-term upper limb impairment, which impacts the quality of life (QOL) and social reintegration, but there is lack of effective therapeutic strategies and novel technologies. Customized multi-muscle functional electrical stimulation (FES) based on the muscle synergy of healthy adults and robotic-assisted therapy (RAT) have been proved efficacy respectively. Synergy-based FES combined with RAT can be a novel and more effective therapy for upper limb recovery of stroke survivors from the perspective of synergistic enhancement. However, few studies have examined the effectiveness of combined synergy-based FES and RAT, especially for motor control evaluated by reach-to-grasp (RTG) movements. The main objective of the following research protocol is to evaluate the effectiveness and efficacy, as well as adoptability, of FES-RAT and FES or RAT rehabilitation program for upper limb function improvement after stroke.

**Methods:**

This will be an assessor-blinded randomized controlled trial involving a 12-week intervention and a 6-month follow-up. Stratified randomization will be used to equally and randomly assign 162 stroke patients into the FES + conventional rehabilitation program (CRP) group, RAT + CRP group and FES-RAT + CRP group. Interventions will be provided in 5 sessions per week, with a total of 60 sessions. The primary outcome measurements will include the Fugl-Meyer Assessment and Biomechanical Assessment of RTG movements. The secondary outcome measurements will include quality of life and brain neuroplasticity assessments by MRI. Evaluations will be performed at five time points, including at baseline, 6 weeks and 12 weeks from the start of treatment, and 3 months and 6 months following the end of treatment. A two-way analysis of variance with repeated measures will be applied to examine the main effects of the group, the time factor and group-time interaction effects.

**Discussion:**

The results of the study protocol will provide high quality evidence for integrated synergy-based FES and RAT, and synergy-based FES alone and guide the design of more effective treatment methods for stroke rehabilitation.

**Trial registration:**

ChiCTR2300071588.

**Supplementary Information:**

The online version contains supplementary material available at 10.1186/s12883-023-03369-2.

## Background

Stroke is the leading cause of long-term disability among middle-aged and elderly adults worldwide [1, 2]. Approximately 70–80% of stroke survivors have limb motor impairments, especially upper limb impairment, which directly impacts the quality of life (QOL) and social reintegration [3]. There is an important effort worldwide to establish therapeutic strategies and novel technologies to improve upper limb function after stroke, such as functional electrical stimulation (FES) [4], robotic-assisted therapy (RAT) [5], and task-oriented therapy (TOT) [6]. Unfortunately, six months after stroke, approximately 65% of patients still cannot incorporate the affected arm and hand into their daily activities [7, 8]. Therefore, the primary goal in the clinical field of upper limb rehabilitation for stroke patients is the continuous exploration of novel and effective treatment therapies.

The central nervous system (CNS) recruits a reduced and fixed set of coordinated patterns of muscle activities to generate a vast variety of movements, which is defined as muscle synergy [9, 10], and several neuroscience studies have demonstrated that muscle synergy is the neurological mechanism of motor execution and control [11, 12]. Compared with healthy controls or the unaffected side, the affected side of stroke patients showed reduced synergy and merged motor modules, which is closely related to poor motor function and motor control [13, 14]. It is likely that this reduction is caused by a lack of independence of the corticospinal drive to the spinal cord, which ultimately results in poor muscle control [15, 16]. Thus, muscle synergy could be a potential ground upon which novel therapies aimed at enhancing motor relearning could be designed, especially for multi-muscle FES therapy.

In this scope, FES training based on muscle synergies in healthy individuals has been recently proposed for the forward-reaching upper limb movements of stroke patients [12, 17, 18]. Specifically, synergy-based FES had instantaneous effects on upper limb motor functions poststroke after 1 session of training, and the potential benefits included increased velocity and motor functionality [11]. The research team of Niu from Ruijin Hospital further verified the short-term (five-day training) efficacy of synergy-based FES for upper limb function based on small samples (9 participants with stroke) [17]. These results demonstrated that synergy-based FES has potential value for improving upper limb motor performance and function. However, the lack of randomized controlled trials with larger sample sizes and evidence of the long-term efficacy of synergy-based FES for upper limb function improvement after stroke might affect its clinical application.

Unlike synergy-based FES, which activates weak muscles, robotic-assisted therapy (RAT) is another upper limb rehabilitation approach that allows for intensive, high-frequency repeat training in accordance with the principles of motor learning [6, 19–21]. More importantly, thus far, the RAT training system could design an accurate movement trajectory that fits activities of daily living (ADLs) as a training target for driving optimal control strategies in real environments, such as reach-to-grasp (RTG) movements. Previous systematic reviews have reported that RAT for the affected upper limb can improve arm-hand performance and health-related quality of life in stroke survivors [6]. Both rehabilitation approaches act on different aspects of motor learning: by means of FES, activation of the neuromotor trajectories is reinforced by exploiting the intentional will of a subject to activate their muscles within a specific task, while through robotic therapy, it is possible to perform a large number of repetitions [21].

Perhaps the best stroke rehabilitation program should combine rehabilitation therapies with definite efficacy on motor function that act on different aspects of motor relearning principles to maximize motor function improvement in stroke survivors [21–23]. It has been proposed that when muscles are activated by electric stimulation and other forms of physical exercise (i.e., RAT, task-oriented training) make voluntary efforts or provide the major interaction effect, a synergistic-enhanced rehabilitation effect may be produced [7, 21]. From this perspective, synergy-based FES combined with RAT may be a novel and more effective therapy for upper limb function enhancement in stroke survivors. However, to our knowledge, few studies have examined the effectiveness and safety of combined synergy-based FES and RAT for upper limb function improvement in stroke patients, especially for motor control in RTG movements. Therefore, the present protocol was designed for an assessor-blinded randomized controlled trial, which will include 3 groups: the FES + CRP group, RAT + CRP group and FES-RAT + CRP group. The purposes of this protocol are as follows:

### Aim 1

To provide high-quality evidence of synergy-based FES for improvement of the upper limb function after stroke.

### Aim 2

To examine the effectiveness and safety of combined synergy-based FES and RAT for upper limb function improvement after stroke.

### Aim 3

To compare the efficacy differences in upper limb function improvement after stroke between the FES-RAT therapy and FES or RAT groups.

## Methods

### Study design

**Fig. 1:**
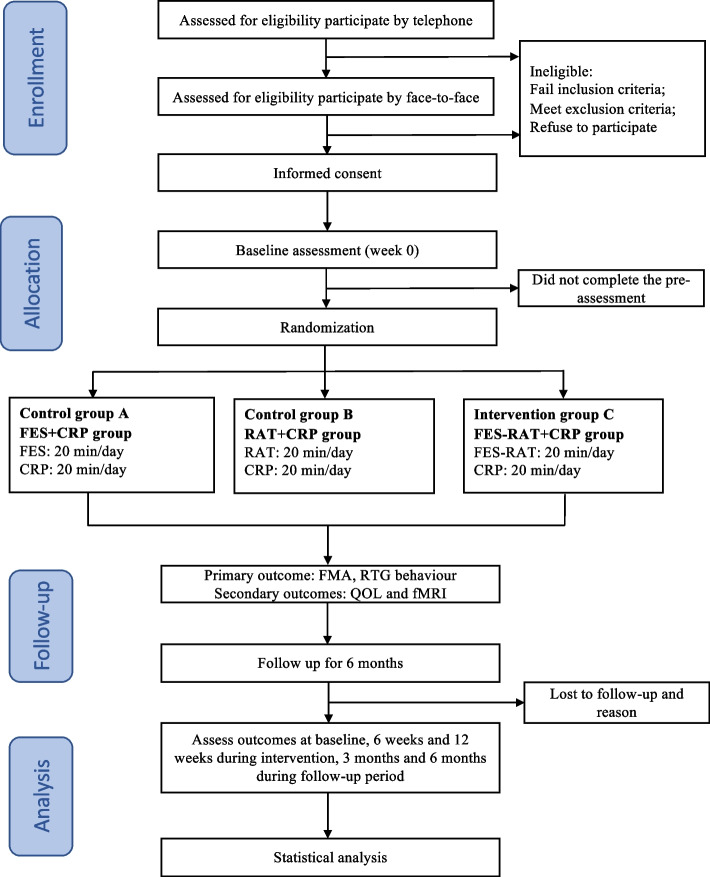
A brief flowchart of the entire study. Note: FES, Functional Electrical Stimulation; CRP, Conventional Rehabilitation Program; RAT, Robotic-Assisted Therapy; FMA, Fugl-Meyer Assessment; RTG, Reach-To-Grasp; QOL, Quality of Life

This will be a single-center, assessor-blinded randomized controlled trial (RCT) involving a 12-week intervention and 6-month follow-up. A total of 162 patients with stroke will be recruited and randomly assigned to the FES + CRP group, RAT + CRP group and FES-RAT + CRP group at a ratio of 1:1:1. Figure 1 depicts a concise flowchart of the entire study, and Table 1 provides the schedule of events. The study protocol (2023-7th-HIRB-034) was approved by the Ethics Committee of the Seventh People's Hospital of Shanghai University of Traditional Chinese Medicine and registered in the Chinese Clinical Trial Registry (ChiCTR2300071588).

**Table 1:**
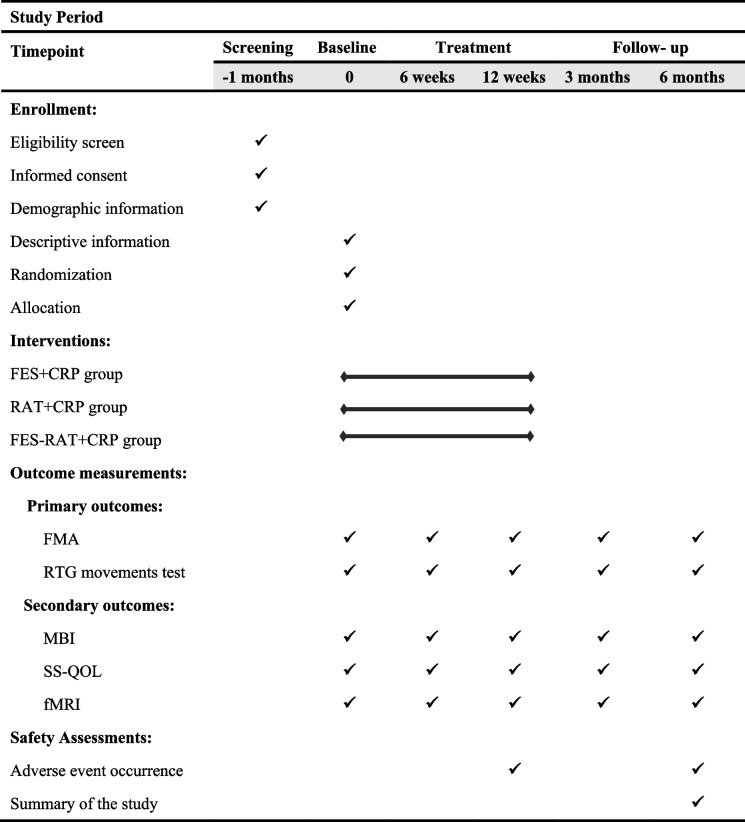
Schedule of enrollment, intervention and assessments

“✓” means the item will be completed. *FES *Functional electrical stimulation, *CRP *Conventional rehabilitation programs, *RAT *Robotic-assisted therapy, *FMA *Fugl-Meyer Assessment, *RTG *Reach-To-Grasp, *BI *Modified Barthel Index, *SS-QOL *Stroke-Specific Quality of Life Scale

### Sample size calculation

The G*power software was utilized to determine the minimum sample size required to detect a significant between-group difference in the present research [24]. In a study of RAT for upper-extremity poststroke rehabilitation, Takebayashi et al. [25] used the minimal clinically significant difference as a reference (4.25 points), the mean difference in the FMA-UE score between patients in the RAT and CRP was 4.50 points and standard deviations between patients were 6. Based on a prior two-way repeated analysis of variance (ANOVA) F test, with a power of 0.80, an effect size of 0.158, three groups, five measurements, correlation among repeated measures of 0.5, and an alpha level of 0.05, 129 participants were required for this study. To account for a conservative drop-out rate of 20%, the final sample size will therefore be 54 participants in each group, for a total of 162 participants.

### Participants

#### Inclusion criteria

The following criteria must be met for inclusion: (1) patients aged between 19 and 80 years old; (2) patients with first-ever stroke, with a time since stroke onset ≤ 3 months; (3) patients with unilateral right or left-sided stroke; (4) patients with a Mini-Mental State Examination score > 24 [26]; (5) patients with at least minimal antigravity movement in the affected upper limb; (6) patients with at least 5 degrees of wrist extension in the antigravity position; (7) patients without hemi-spatial neglect; and (8) patients who sign the informed consent form.

#### Exclusion criteria

The following criteria will result in exclusion: (1) any active uncontrolled medical, cardiovascular, or orthopedic condition; (2) significant upper limb peripheral neuropathy; (3) severe shoulder pain or wrist and finger contractures; (4) contraindication to FES, such as a skin allergy; and (5) currently participation in any other clinical study or coach-directed exercise program.

### Setting and recruitment

A total of 162 individuals who comply with the inclusion criteria will be recruited from the Seventh People's Hospital of Shanghai University of Traditional Chinese Medicine and neighboring communities using flyers, posters, and referrals from a rehabilitation therapist or neurologist. The participants will be invited to undergo an in-person examination and evaluation to guarantee that they satisfy the inclusion criteria and fail to meet the exclusion criteria. In addition, we will inform all participants of the study's purpose, methodology, and prospective benefits, as well as the principle of voluntary participation. All participants will be asked to provide written informed consent. Recruitment began on 1 May 2023 and will continue until 162 individuals are enrolled.

### Randomization, allocation concealment and blinding

To minimize any potential bias, the eligible participants will be randomly assigned to 3 groups using stratified randomization with sex (male/female), stroke type (hemorrhage/infarction) and dominant hand (left/right) as factors [27]. After categorization, there will be eight subgroups: (1) male, hemorrhage, left hand dominant; (2) male, hemorrhage, right hand dominant; (3) male, infarction, left hand dominant; (4) male, infarction, right hand dominant; (5) female, hemorrhage, left hand dominant; (6) female, hemorrhage, right hand dominant; (7) female, infarction, left hand dominant; (8) female, infarction, right hand dominant. For any one subgroup, the participants will be randomly divided into groups A, B, and C; then, all A, B, and C groups will be merged to form new group A, B, and C groups. The randomization number lists will be generated by the random number generator of SPSS software (IBM Corp. Released 2013. IBM SPSS Statistics for Windows, Version 22.0. Armonk, NY: IBM Corp.).

The allocation of participants will be secured in opaque numbered and sequentially sealed envelopes, which will be prepared by an independent researcher. The corresponding envelopes will be opened to ascertain a patient’s group assignment after all baseline assessments have been completed by the participants. In addition, physiotherapists with the same level, robotic and FES operators will also follow randomization and be randomly assigned to one of the groups.

The outcome assessors and statisticians who implement the final statistical analyses will be blinded to avoid any potential detection bias.

### Intervention

The rehabilitation intervention will be administered at the rehabilitation medical center of the Seventh People’s Hospital of Shanghai University of Traditional Chinese Medicine. All participants will receive the CRP recommended by the neuro-rehabilitation guidelines [28, 29], which will include stretching, passive and active limb movements, proprioceptive neuromuscular facilitation, muscle strengthening, and different reaching, grasping and manipulating actions that are motivational and appropriate for the participants. Based on the CRP (20 min), Group A will receive FES (20 min); Group B will receive RAT (20 min); and Group C will receive FES-RAT (20 min). The intervention for the three groups will be provided for 60 sessions (5 sessions per week, 12 weeks), each lasting 40 min.

### Functional electrical stimulation (FES)

A synergy-based multi-channel FES device was developed using the Fourier ElectroFortis Programmable Stimulator, which can stimulate multiple muscles with customized parameters. An experimenter computer GUI application was created to configure the following basic stimulation parameters: the frequency, minimum and maximum intensity, pulse duration, ramp time, synchronization and order of stimulations, type of user interactions and number of repetitions [30]. The stimulation envelope will be specified as the concatenated piecewise linear functions (e.g., rising phase, plateau phase and falling phase), and will be allowed to be preloaded to the FES device [11]. Synergy-based FES will be applied to stroke participants and constructed from the muscle synergies of healthy controls. The raw EMG signals of the dominant upper limb muscle will be obtained from healthy controls performing the RTG task [31]. After processing with a filter and rectification, the muscle synergy pattern will be extracted by non-negative matrix factorization (NMF) in the previous study [32]. Then, the muscle synergy pattern will be converted into the FES stimulus envelope according to the conversion formula proposed by Niu [11].

**Fig. 2:**
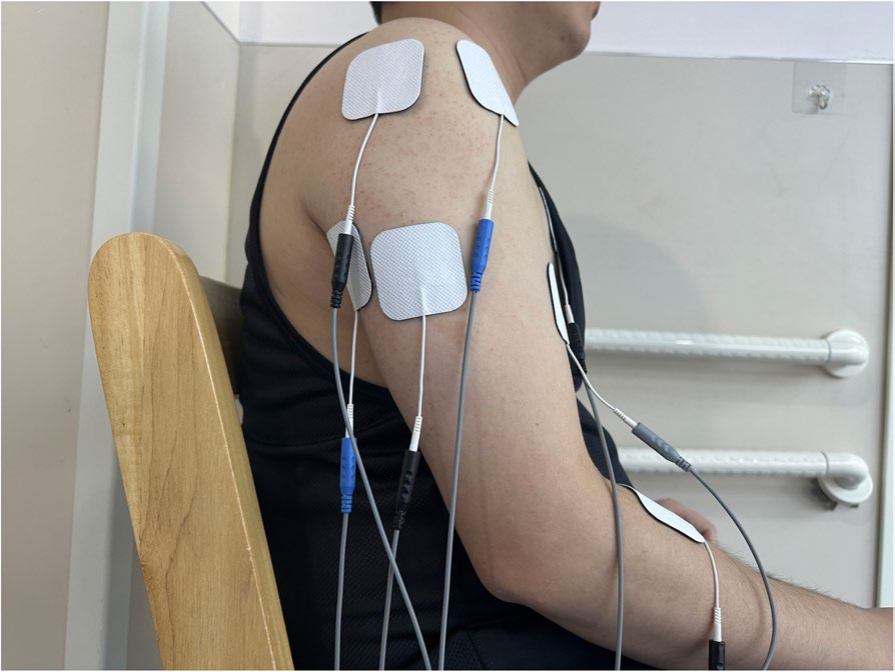
The points of the identified motor muscles in FES

The training objective for FES therapy emphasizes functional arm reaching, hand opening and grasping. During RTG training, gross motor tasks (proximal muscles) are started in the early stages of therapy, followed by fine movement control tasks (distal muscles) [30]. As participants improve, stimulation will gradually be reduced to a minimum and eventually phased out. Before the intervention, all operators will receive the homogenization training of the FES treatment scheme. The specific steps are as follows:

1. Identify the functions to be trained and select the order of the tasks to be retrained.
2. Identify the stimulated muscle and apply self-adhesive electrodes over the identified motor muscles: including the biceps brachii, triceps brachii lateral head, triceps brachii long head, anterior deltoid, posterior deltoid, pectoralis major, and brachioradialis [33] (Fig. 2).
3. Identify and record the different stimulation thresholds: • the motor threshold: when a palpable or a visible contraction is produced; • the sensory threshold: when the participants feel the current for the first time; and • the maximum threshold: beyond which the patient does not tolerate an increase in current amplitude.
4. Preloaded the muscle synergy pattern of the healthy controls to the FES device.
5. Explain what to expect when FES is started and instruct the participants to make an active attempt to perform the intended movement.
6. Repeat the stimulus protocol and ensure an adequate rest time.
7. Turn off the stimulator when the treatment is completed, remove the electrodes and inspect the skin underneath for any redness.

### Robotic-assisted therapy (RAT)

The Fourier intelligence upper-limb robotic system (Fourier, Shanghai, China), which is an intelligent rehabilitation training system designed especially for stroke patients with upper limb or motor dysfunction, will be used for robotic-assisted therapy in this study. The system can provide passive, assistive, and active planar movements around the shoulder, elbow, and wrist joints [31]. More importantly, the system is able to establish the movement trajectory of the manipulator according to the upper limb movement pattern during ADLs or target tasks, which can be customized for specific tasks. The largest body of clinical evidence has been amassed for this robotic system; the system has been successfully tested on over 500 stroke patients in clinical studies, and there are approximately 100 robots in use worldwide [34].

**Fig. 3:**
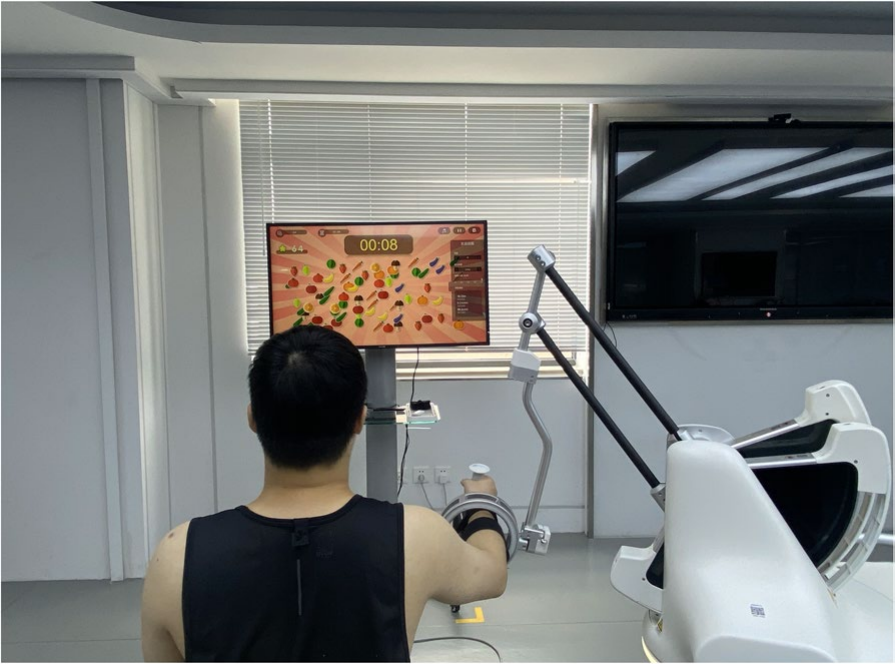
RAT tasks for upper limb

During treatment, participants will perform the RTG task displayed in a game scene that generates real-time auditory and visual feedback [35] (Fig. 3). Each participant will be required to sit on a chair, and his or her affected UE will be strapped to the robot arm. Machine settings such as the height of the platform, the length of the robot arm, and the amount of assistance force will be adjusted according to each participant’s personal characteristics. The treatment content (game tasks/motor or task-oriented training) settings and doses will be the same for all participants in the middle activity range. RAT will provide repetitive and high-intensity training in a cost-effective manner. When participants perform the RTG movement, the objects will be exhibited on the computer screen using a virtual hand, allowing them to receive timely feedback on their performance from the robotic device. The robot’s UE movement patterns are functional and the joints are isolated, which minimizes synergic movement in stroke patients.

### FES-RAT

**Fig. 4:**
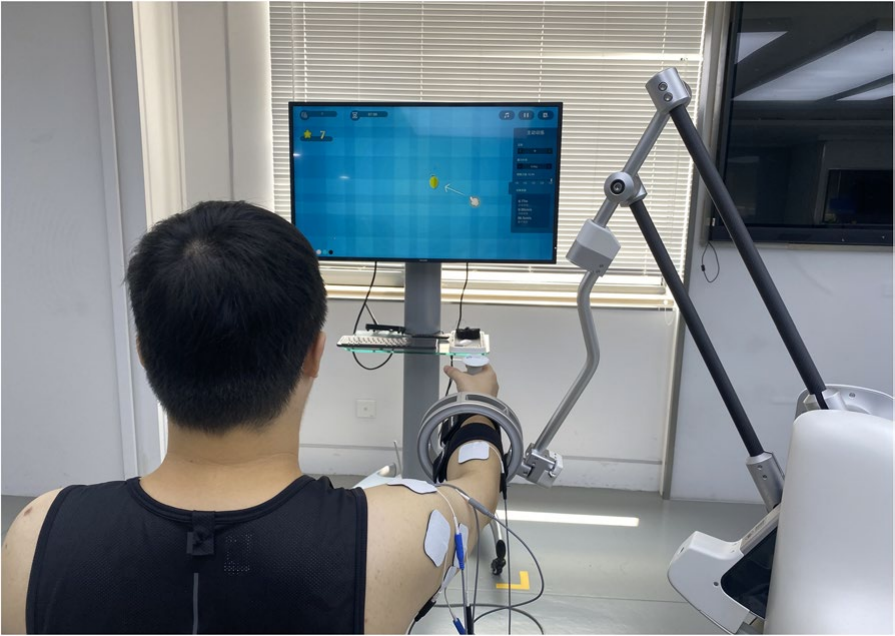
FES combined RAT in upper limb training

For FES-RAT treatment, the participants will receive FES when performing the RAT task (Fig. 4). The frequency, repeated time and rest time will be consistent with separate FES or RAT. To realize the synchronous triggering and control of FES and RAT, the control terminal will be developed through a multifunctional I/O Device (NI6255, National Instruments Inc., Austin, TX) [36]. The FES stimulus envelope and manipulator kinematic datasets will be aligned via the start switch trigger recorded digitally in HANDoVR, and the analog reading of the digital output will be sent from HANDoVR to MATLAB.

### To reduce the dropout rate

To improve treatment adherence and reduce the likelihood of participants dropping out of the study, the therapists will contact the participants by texting, voice and video through an intelligent follow-up system, which is a small program embedded in WeChat app. The therapists will also regularly phone the participants if they have difficult in receiving information on smartphones. Through above strategies, the research team can confirm their appointments, discuss the subsequent intervention step as well as avoiding any barriers to adherence.

### Outcome measurements

All outcome measurements will be assessed by a professional rehabilitation assessor who will be blinded at baseline (T1), 6 weeks (T2) and 12 weeks (T3) during the intervention and at 3 months (T4) and 6 months (T5) during the follow-up period. Data on the demographic and clinical characteristics of the participants will be collected at T1.

### Primary outcome measurements

#### Upper extremity function

The primary outcome is upper extremity function evaluated by the upper extremity section of the Fugl-Meyer scale. The FMA-UE has outstanding intrarater (ICC = 0.997) and interrater (ICC = 0.993) reliability for assessing proximal-to-distal and synergistic-to-isolated movement behavior in stroke patients [37, 38]. The highest possible total score is 66, with 33 items and ordinal scoring from 0 to 2, with a higher score indicating more effective function. Additionally, both the proximal (range 0–42) and distal (range 0–24) subscores of the FMA-UE will also be considered.

#### Biomechanical assessment of reach-to-grasp movements

The RTG movements of the affected upper extremity will be assessed by three-dimensional biomechanical analysis. The RTG performance test will be selected because prior research has demonstrated that concentrating purely on task outcome measures fails to differentiate between neural recovery processes and the development of efficient but abnormal compensatory movement patterns [39–42]. Multiple authors have utilized the kinematic analysis of three-dimensional RTG movements as a viable means to analyze the motor control process and identify the normalization of motor function in persons with stroke [40, 42, 43]. The biomechanical evaluation protocol will be based on a previously established, standardized kinematic analysis testing protocol for RTG movements (i.e., drinking) [40, 42]. More comprehensively, the present study protocol will further complement the evaluation of muscle activation and synergy patterns.

The standardization of the RTG task will be divided into five phases: (1) reaching to grasp a glass, (2) forward transport of the glass to the mouth, (3) drinking a sip of water, (4) transporting the glass back to the table, and (5) returning the hand to the initial position [41]. The details of the test task are as follows: the participants will sit comfortably at a table, with 90° knee and hip flexion and 90° elbow flexion with the upper arm vertical and forearm horizontal. The grasping target will be rigidly mounted on the testing table in front of the subject, aligned with the participant’s affected shoulder and at a distance of 35 cm. Data from each participant will be collected from 5 successful trials, and the rest time between each trial will be approximately 30 s.

Before the biomechanical test, the reflected markers and EMG sensors will be placed on the anatomical landmarks and targeted muscle respectively. Eight markers will be affixed on the tested hand (III metacarpophalangeal joint), the wrist (styloid process of the ulna), the elbow (lateral epicondyle), on both shoulders (acromion), the trunk (sternum), the forehead and the drinking glass. EMG sensors will be placed on the following ten muscles of each participant’s shoulder, arm and hand on the affected side: the first dorsal interosseous (FDI), flexor digitorum superficialis (FDS), extensor digitorum communis (EDC), extensor indicis (EI), abductor pollicis brevis (APB), extensor pollicis brevis (EPB), biceps brachii (BB), triceps brachii (TB), anterior deltoid (AD), and posterior deltoid (PD). A 10–camera infrared motion capture system (sampling rate: 100 Hz; Vicon Motion Systems, Oxford, United Kingdom) and a Delsys EMG system (sampling rate: 1000 Hz; Delsys Inc., Natick, MA) will acquire three-dimensional kinematics and muscle activation information, respectively.

The outcomes of kinematic analysis will include the following: the movement time (ms), peak velocity (cm/s), time to peak transport velocity (ms), trajectory length ratio (%), trajectory smoothness, peak aperture (cm), peak aperture velocity (cm/s), opening distance (cm) and reach grasp coupling index. The definitions of kinematic variables have been described in several previous studies [36, 40, 42–44]. Additionally, the muscle synergy pattern will be extracted from the raw EMG signals through the method of non-negative matrix [11, 32], which considers muscle activation as the linear combination of multiple muscle components (muscle vectors) with the corresponding activation coefficients (time profiles).

### Secondary outcome measurements

#### Quality of life

The QOL of the participants will be measured by the Stroke-Specific Quality of Life Scale (SS-QOL). The SS-QOL was created in 1999 to quantify the impacts of stroke and assessed the efficacy of treatment strategies with high reliability (0.98) [39, 40]. The SS-QOL is a long 49-item stroke-specific instrument that provides a total QOL score and 12 subscale scores associated with mobility, cognition, mood, functionality, and social roles, with higher scores indicating a better QOL.

#### Brain neuroplasticity

Magnetic resonance imaging (MRI) will be used to investigate the brain mechanisms of rehabilitation, particularly in regions related to motor learning and motor control. MRI data will be acquired using a 3.0 T magnetic resonance scanner (Siemens Magnetom Verio Syngo MR B17, Germany) with a 32-channel phase-array head coil at the Department of Medical Imaging of the Seventh People's Hospital of Shanghai University of Traditional Chinese Medicine. The following three MRI modalities will be acquired [41–43]: (1) high-resolution anatomical MRI (T1-MRI) for estimating the anatomical parameters of the cortex, the subcortex, and structural connectivity; (2) resting-state functional MRI (RS-fMRI) for estimating the functional connectivity of brain regions; and (3) diffusion tensor imaging (DTI) for estimating the tract-graphic parameters of white matter and the microstructure of gray matter.

### Data management and monitoring

The project and data management, data analysis and data monitoring will be supervised by the independent Data Monitoring Committee (DMC) affiliated with the Scientific Research Innovation Platform of the Seventh People's Hospital of Shanghai University of Traditional Chinese Medicine. The dataset will be stored, analyzed and archived in a pseudonymized manner to entirely protect individual privacy and minimize bias.

### Statistical analysis

All statistical analyses will be performed with IBM SPSS (IBM Corp. Released 2013. IBM SPSS Statistics for Windows, Version 22.0. Armonk, NY: IBM Corp.) by statisticians who are blinded to the group allocation. The analyses will be performed on an intention-to-treat basis. The Shapiro–Wilk (SW) test will be used to examine the normal distribution of the continuous variables comprising demographic and outcome measures. Continuous variables will be described as the mean ± SD for those with normal distributions or the median for those with nonnormal distributions, and categorical variables will be described as the frequency. The χ^2^ test or Fisher’s exact test will be used to examine the comparisons among the three groups for categorical variables. When the normality of data distribution is determined, two-way of variance with repeated measures will be applied to examine the main effects of the group and time factors, as well as the group-time interaction effects. A simple effect post hoc analysis will be conducted when the time-group interaction is significant. Furthermore, the linear mixed model will be adjusted for age, sex and type of stroke if homogeneity is not found. The significance level for all statistical tests will be set at 0.05, corrected for multiple comparisons using the Bonferroni-adjusted method and accompanied by a 95% confidence interval.

### Ethics and dissemination

This study procedure will be conducted in accordance with the principles of the Declaration of Helsinki in its current version (for details, see www.wma.net). Ethics approval (2023-7th-HIRB-034) was granted by the Research Ethics Committee of the Ethics Committee of the Seventh People's Hospital of Shanghai University of Traditional Chinese Medicine. The results of the study will be published in peer-reviewed scientific journals and presented at conferences and workshops after study completion.

## Discussion

The recovery of motor function is an important goal in the rehabilitation of poststroke patients [7]. Customized multi-muscle functional electrical stimulation (FES) based on the muscle synergy of healthy adults may assist in improving upper limb motor function and motor control in stroke patients [12, 17, 18]. However, relevant studies have only reported the instantaneous and short-term effects of synergy-based FES. Synergy-based FES combined with robotic-assisted therapy (RAT) can be a novel and more effective therapy for upper limb function improvement of the stroke survivors, but few studies have examined its effectiveness, especially motor control in the RTG movement. Based on the above problems, the authors have designed a comprehensive research scheme framework including a 12-week intervention and a 6-month follow-up, which allows three study purposes to be realized simultaneously.

This study has several important strengths. First, it is a novel integrated treatment method for combining synergy-based FES and RAT interventions, based on integrated rehabilitation technologies and synergistic enhancement effects for improving the upper limb function after stroke. Previous studies examined individual components (e.g., CRP, FES, RAT) but have not combined them in an integrated method [4, 20, 29]. Second, the comprehensive intervention protocol will be evidence-based and rigorously developed based on the evidence, recommendations, theories and practice standards of the systematic review [4, 6]. Third, more systematic and comprehensive assessment outcomes will be selected for this protocal based on biomechanical and brain science, such as the biomechanical assessment of RTG movements (e.g., kinematics, EMG), and resting-state functional MRI (RS-fMRI) for estimating the functional connectivity of brain regions [44, 45]. Therefore, the present study will provide a more comprehensive and systematic protocol for the further study with a randomized controlled trial design in the stroke rehabilitation.

We recognize that this study also has limitations. This research has an inevitable limitation associated with the difficulty in controlling the methodology of blinding during the RAT intervention because the participants and therapists cannot be blinded due to the visibility of the RAT intervention. Although the outcome assessors and data analyzer will be blinded to the group allocation, there might still be a risk of detection bias during the study’s implementation. Additionally, muscle synergy extraction, FES and RAT training in this study need to be performed by medical personnel in medical institutions, which may limit the promotion and application of synergy-based FES and RAT in the community and home settings. Furthermore, based on the results of a few recent studies, there were no differences between intracerebral hemorrhage and cerebral infarction of function prognosis at rehabilitation discharge, so when including patients, we didn’t make a clear distinction on whether different types of strokes will affect the intervention outcomes, which should be investigated in further study.

In summary, the results of the study protocol will achieve multiple study purposes, including demonstrating the effectiveness of synergy-based FES, and synergy-based FES-RAT for improving upper limb function after stroke and exploring the efficacy differences in improving upper limb function after stroke between the FES-RAT therapy and FES or RAT. Synergy-based FES combined with RAT may have a potential opportunity to better improve the upper limb function of stroke survivors. These results of the study will provide high-quality evidence for integrated synergy-based FES and RAT and synergy-based FES alone and guide the design of more effective treatment methods for stroke rehabilitation.

### Supplementary Information

**Additional file 1.** SPIRIT 2013 Checklist: Recommended items to address in a clinical trial protocol and related documents*.

## Data Availability

Not applicable.

## Abbreviations

FES: Functional Electrical Stimulation
RAT: Robotic-assisted Therapy
RTG: Reach-To-Grasp
CRP: Conventional Rehabilitation Programs
QOL: Quality of Life
TOT: Task-oriented Therapy
CNS: Central Nervous System
RCT: Randomized Controlled Trial
ADL: Activity of Daily Living
NMF: Non-negative Matrix Factorization
FDI: First Dorsal Interosseous
FDS: Flexor Digitorum Superficialis
EDC: Extensor Digitorum Communis
EI: Extensor Indicis
APB: Abductor Pollicis Brevis
EPB: Extensor Pollicis Brevis
BB: Biceps Brachii
TB: Triceps Brachii
AD: Anterior Deltoid
PD: Posterior Deltoid
MRI: Magnetic Resonance Imaging
RS-fMRI: Resting-state Functional MRI
DTI: Diffusion Tensor Imaging
DMC: Data Monitoring Committee
FES: Functional Electrical Stimulation
